# Interrelationships Between Psychosocial, Motivational, and Psychological Processes for Effective Learning: A Structural Equation Modeling Study

**DOI:** 10.3389/fpsyg.2021.740965

**Published:** 2021-09-30

**Authors:** Huy P. Phan, Bing H. Ngu

**Affiliations:** School of Education, University of New England, Armidale, NSW, Australia

**Keywords:** psychological processes, motivation, adaptive outcomes, schooling experience, optimal best, perceived social experiences, positive psychology, optimization

## Abstract

We tested a theoretical-conceptual model that introduced our recently developed psychological concept, termed as *psychological processes*, which is defined as “a person’s continuing frame of mind to focus on disposition toward strong resolute, structured thoughts and organization, and aspiration to strive for educational success.” This proposition is innovative as it considers the notion that a person’s mindset is malleable and, importantly, subjects to social experiences derived from a situated social context. Moreover, from our definition, we contend that psychological processes, as a distinct construct, is “latent,” or underlying, with three comparable psychological attributes: personal resolve, effective functioning, and personal striving. Our conceptualization, acknowledging the importance of social contexts and individualized experience and personal belief, proposed that perceived social experiences (i.e., positive versus negative), as a source of information, would shape a student’s psychological processes, his/her state of motivation, and engagement in different types of adaptive outcomes. Moreover, from our point of view, psychological processes would act as a predictor as well as a potential mediator of motivation and engagement in different types of adaptive outcomes. In a similar vein, from the positive effect of psychological processes, motivation could act as a predictor as well as a mediator of adaptive outcomes. Structural equation modeling, from Taiwanese university students’ (*N* = 739) responses to various Likert-scale measures, showed support for our original *a priori* model – for example, the positive effects of perceived social experiences on psychological processes (β = 0.81, *p* < 0.001) motivation (β = 0.61, *p* < 0.001), and adaptive outcomes (β = 0.36, *p* < 0.01), and the positive effect of psychological processes on motivation (β = 0.31, *p* < 0.01). Interestingly, we also found some interesting findings with regard to the effects of measured indicators – for example, the positive effect of personal resolve, as a measured indicator, on adaptive outcomes (β = 0.28, *p* < 0.001), and the effect of self-efficacy, as a measured indicator, on academic liking experience, also a measured indicator (β = 0.12, *p* < 0.01). Overall, the results established have a wide range of implications for consideration – for example, the development of an educational program and/or instructional design that could promote and foster positive learning experiences.

## Introduction

One notable research inquiry in the field of Educational Psychology has emphasized the importance of effective learning and enriched schooling experiences. This focus, interestingly, has involved researchers and educators using different theoretical orientations as grounding for their conceptualizations. Recently for example, drawing from the *paradigm of positive psychology* (Seligman, 1999, 2010; Seligman and Csíkszentmihályi, 2000), we developed the *theory of human optimization* (Phan et al., 2017b, 2019b, 2020b), which seeks to provide theoretical understanding into the dynamics of “optimal best” (Fraillon, 2004; Phan et al., 2016c). Optimal best, reflecting positive schooling experiences, emphasizes a person’s fullest capability in a subject matter (i.e., the maximization of a student’s cognitive functioning in a subject matter in school). How a student achieves optimal best and/or effective learning in school contexts is a pervasive question that is noteworthy for consideration.

Achieving optimal schooling experiences, we contend, requires some form of “optimization” or motivation (Phan and Ngu, 2019a). For example, in a recent study that involved secondary school students (Phan and Ngu, 2021a), we found that a psychological concept known as “personal resolve” (Phan et al., 2017b, 2018, 2018b) actually accounted for an improvement in optimal best. In a study that consisted of university students (Phan et al., 2019d), likewise, we found that aside from personal resolve, three other constructs also predicted optimal best: motivation toward learning, current level of best practice, and proactive social relationship. Thus, from this brief introduction of our current research undertakings and in tandem with existing research inquiries in the area of *student motivation* (e.g., Diseth, 2011; Martin et al., 2013; Collie et al., 2015), we contend that there are comparable and comparative psychological models, which may have practical relevance and explain the dynamics of optimal best and effective learning. Our collective research interest has led to our continuing development of different conceptualizations for investigation (e.g., Phan et al., 2019d, 2020e; Phan and Ngu, 2021a), which in turn could advance the study of optimal best (Fraillon, 2004; Phan et al., 2016c, 2017b). One such conceptualization, as reported in the present empirical article, capitalizes on the use of the statistical technique of *structural equation modeling* (SEM) (Schumacker and Lomax, 2004; Kline, 2011).

**FIGURE 1:**
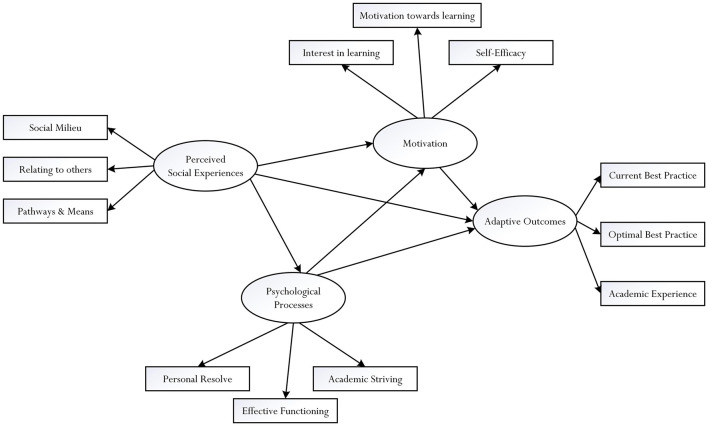
Conceptual model for examination.

The significance of our research inquiry, as discussed in this empirical article, entails an examination of a theoretical model (see Figure 1), which considers the interrelationships between three major theoretical orientations of effective learning: *psychosocial experiences*, *psychological processes*, and *motivational beliefs*. Specifically, our focus of inquiry, which consists of university students in Taiwan (*N* = 442 women, 297 men), entails two different structural pathways of analysis: the hypothesized *a priori* effects of four distinct latent theoretical concepts, denoted as L_1_ predictive effects, and the potential *a posteriori* effects of the corresponding measured indicators, denoted as L_2_ predictive effects. This consideration of both L_1_ and L_2_ predictive effects is insightful, theoretically and methodologically, allowing us to have clearer understanding into the operational nature of psychosocial, psychological, and motivational factors of students’ learning. More importantly, aside from theoretical and methodological contributions, our obtained correlational findings are of relevance for educators and students alike – for example, the effective utilization of psychological processes, which may facilitate the achievement of optimal best.

## An Integrated Framework for Development: Introduction

Fostering enriched learning experiences in academic contexts is an important endeavor for development. In the field of research of Educational Psychology, for example, educators and researchers have inquired into different theoretical orientations that could promote and account for effective learning. Capitalizing on this development, we conceptualize a theoretical model, which incorporates three major orientations for examination: *psychosocial influences*, *psychological processes*, and *motivational beliefs*. This proposition for examination, as shown in Figure 1, is situated within the framework of structural equation modeling (SEM) (Schumacker and Lomax, 2004; Kline, 2011), which consists of a latent factor (i.e., a latent factor is known as ξ_*n*_, where *n* = 1, 2, 3,….) and its defined measured indicators (i.e., a measured indicator is known as X_*n*_, where *n* = 1, 2, 3,….). Our conceptualization connotes three distinct latent factors, corresponding to the three major theoretical orientations: psychosocial influences (ξ_1_), psychological processes (ξ_2_), and motivational beliefs (ξ_3_). Each latent factor is defined by three measured indicators (e.g., ξ_1_ → X_1_, X_2_, and X_3_) where, in turn, each measured indicator is a composite score of item responses. This methodological approach is significant as it enables us to examine and identify associative patterns of both latent factors (i.e., L_1_ predictive effects) and measured indicators (i.e., L_2_ predictive effects). For clarity, Table 1 summarizes the definition and the description of the nature of each measured indicator. In the next section of the article, we provide an overview of the potential relationships between the three latent factors and their respective measured indicators.

**TABLE 1:** A summary of definitions.

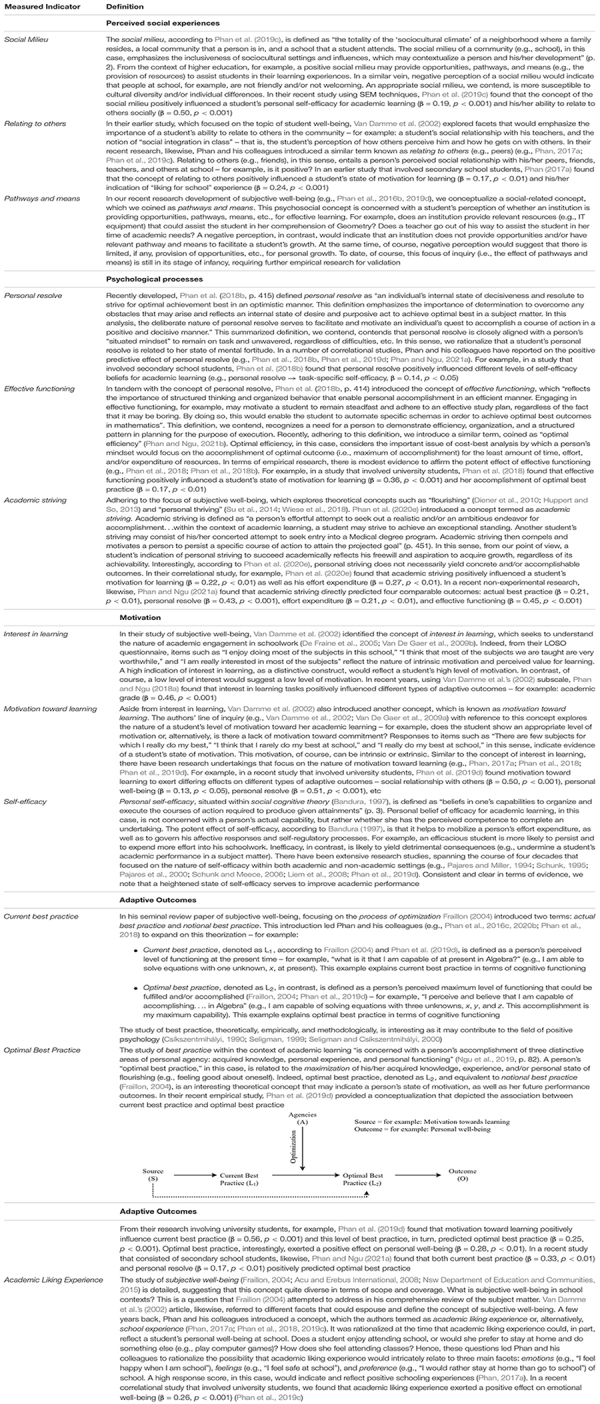

*Information presented in Table 1 is summarized in a concise manner to showcase the proposed concepts and their significant nature. The presentation of the information is not intended to provide comprehensive understanding, especially in terms of coherence and conceptualization.*

### The Importance of Perceived Social Experiences

There is extensive research, which has focused on the importance of the social context. Vygotsky (1978) *sociocultural theory of cognition*, for example, places emphasis on the social processes – that a person, for example, would internalize his learning and understanding about Psychology 101 from peers and capable others. Bronfenbrenner (1989) *bioecological systems theory*, similar to Vygotsky (1978) theory, discusses the importance of situated sociocultural influences. Roorda et al. (2011) meta-analysis of engagement, likewise, details the impact of the social environment. According to the authors, a social environment is perceived as being positive when opportunities arise for proactive social relationships (e.g., teacher-student social relationship: Hawkins et al., 2010; Allen et al., 2013; Gallagher, 2013). Why is this the case? A student is more inclined to have a favorable view when a teacher or an educator is able to provide social support and emotional security. This premise, in part, reflects the nature of *attachment theory* (Ainsworth et al., 1978; Ainsworth, 1979; Stevenson-Hinde and Verschueren, 2002), which emphasizes a student’s need to seek emotional and social bonding.

From the perspective of schooling and academia, it is poignant for students to perceive positive social experiences. Perceived enriched social experiences, such as a positive teacher-student relationship (TSR) (Roorda et al., 2011) would assist to facilitate school, or academic, adjustment, resulting in improved academic and non-academic performances. In the context of the present study, we define a student’s perceived social experiences, ξ_1_, as being a composite, or an amalgamation, of the following: the student’s perception of the social milieu itself (X_1_), the student’s ability to socially relate to others at school (X_2_), and the student’s perception that there are pathways and means for academic growth (X_3_). Positive social experience at school (e.g., supporting friendship with a peer) is effective (Roorda et al., 2011; Whannell and Allen, 2011) as it helps students to adjust and to improve their academic performance outcomes. For example, in line with Vygotsky (1978, 1981) *sociocultural theory of cognition*, a student may actively interact and seek academic support from a teacher and/or from a peer who is more capable in the subject matter.

Aside from proactive social relationships at school, opportunities, pathways, and/or means for personal growth in academic learning and/or personal well-being (Li et al., 2008; Soutter, 2011; Waters et al., 2017) are also encouraged. For example, in a recent study, which involved university students, Phan et al. (2019c) found that relating to others accounted for an improvement in positive emotional well-being. In a similar study that consisted of secondary school students in Taiwan, Phan and Ngu (2020a) noted that proactive social relationship between teachers and students predicted personal and positive emotional well-being. Peer-peer social relationship, in contrast, predicted personal well-being and personal striving. From our point of view, taking into consideration this line of evidence, positive perceptions of social experiences (e.g., a friend’s willingness to assist) could help to instill motivation for academic learning, whereas negative perceptions (e.g., a student’s perceived sense that she is not being accepted by other students) would demotivate and serve to weaken one’s resolve to engage.

There is also existing research development, which contends the potent influences of different aspects of perceived social experiences on different sub-psychological processes. For example, in a correlational study that focused on academic engagement, Liem and Martin (2011) found that peer relationships (e.g., opposite-sex relationship at school) positively predicted general self-esteem. In a study that consisted of children with dyslexia, Shehu et al. (2015) found a positive association between social relationships and self-esteem. In one of the earlier studies in the late 1990s, Wentzel (1998, p. 202) using multiple regression analysis reported some interesting patterns: peer support was a positive predictor of prosocial goal pursuit, teacher support was a positive predictor of both types of interest and of social responsibility goal pursuit, and parent support was a positive predictor of school-related interest and goal orientations. More recently, as indicated in Table 1, Phan and his colleagues observed the positive effects of perceived social experiences – for example: the effect of relating to others on personal striving (Phan et al., 2019d), the effect of the social milieu on self-efficacy (Phan et al., 2019c), and the effect of peer-peer social relationship on personal striving (Phan and Ngu, 2020a).

Analysis of existing research suggests then that there is clear and consistent evidence, which affirms the central role of perceived social experiences as an antecedent of different types of adaptive outcomes (Wentzel, 1998; Roseth et al., 2008; Umberson and Montez, 2010; Raufelder et al., 2013; Shehu et al., 2015). This proposition is poignant as it emphasizes the explanatory nature of perceived social experiences, which have both positive or negative connotations – for example: I feel that this school is not supporting me at all and that, importantly, people here are not too “welcoming” (i.e., this is a negative perception of social experience). A positive social experience, in this analysis, is more potent as it would help students cope with their academic learning, seek emotional and/or social support, and develop enriched well-being experiences (e.g., positive emotional well-being). By the same token, of course, there is credence to contend that a person’s perceived social experiences are intricately linked to his/her sub-psychological processes for effective learning (e.g., a person’s self-belief). In the context of the present study, we postulate that perceived positive social experiences in educational contexts (e.g., the perception that there are opportunities and pathways for personal growth) would positively influence different types of psychological and educational variables.

### The Importance of Psychological Processes

One notable outcome of perceived positive social experiences is the enactment of internal psychological processes, ξ_2_, which espouse three comparable sub-processes: personal resolve (X_1_), effective functioning (X_2_), and academic striving (X_3_). This proposed psychological processes concept, from our conceptualization, coincides with the *paradigm of positive psychology* (Seligman and Csíkszentmihályi, 2000; Seligman et al., 2009; Seligman, 2010), which focuses on the notion of “positivity and proactivity” of human agency. One notable distinction of positive psychology, in particular, relates to the understanding that there are sub-psychological processes (e.g., a state of resilience) that may facilitate a person’s state of functioning. For example, within the context of academic learning, there is evidence to indicate the positive effects of positive psychological sub-processes such as a state of buoyancy (Martin et al., 2013; Collie et al., 2015).

Our conceptualization considers three comparable constructs, which could potentially optimize a person’s state of cognitive functioning: personal resolve, effective functioning, and academic striving. An analysis of the information detailed in Table 1 suggests that, in general, personal resolve, effective functioning, and academic striving are similar in terms of their nature and characteristics. One notable distinction in similarity lies in the facilitation and enhancement of a person’s positive state of functioning, such as his/her achievement of optimal best in an academic subject matter (Fraillon, 2004; Martin, 2006; Phan et al., 2016c). For example, research development has noted consistent evidence, which shows the positive effect of personal resolve on the achievement of optimal best (Phan et al., 2019d; Phan and Ngu, 2021a). This line of empirical validation contends that a state of decisiveness and unwavered focus without any uncertainty is likely to associate with and/or instill a perceived of confidence, resulting in a person’s self-determination and subsequent performance outcome. Indecisiveness, in contrast, is more aligned with a person’s sub-optimal experiences, reflecting in his/her underachievement in a subject matter.

Examining the nature of sub-psychological processes of personal resolve, effective functioning, and academic striving, which may operate in tandem with each other, is insightful as this would provide clarity into their combined and/or individual predictive effects on different types of adaptive outcomes. Specifically, as shown in Figure 1, it is plausible to consider two contrasting explanatory and predictive effects: the predictive effect of the latent representation of psychological processes on an adaptive outcome [i.e., ξ_2_ → O, where ξ_2_ = psychological processes, O = adaptive outcome (e.g., a state of motivation), → = predictive effect] versus the predictive effects of the three measured indicators on the same adaptive outcome [e.g., X_1_ → O, where X_1_ = personal resolve, O = adaptive outcome (e.g., a state of motivation), → = predictive effect]. This consideration, we contend, reflects methodological innovation, providing a basis for us to gauge into comparative predictive effects of psychological processes. By the same token, structurally, it is also possible for us to test and identify different types of information, which could assist to account for the formulation of personal resolve, effective functioning. For example, in a recent study that involved university students, Phan et al. (2018) found that a student’s current level of knowledge and understanding of a subject matter, termed as “realistic best practice,” would positively influence a state of effective functioning and his/her personal resolve. Interestingly, with secondary school students, Phan and Ngu (2020a) noted that the formulation of academic striving, defined as a person’s effortful attempt to seek out a realistic and/or an ambitious endeavor for accomplishment, is shaped by different psychosocial factors – academic self-efficacy, global self-esteem, peer-peer social relationship, personal well-being, and belief of optimal best.

### The Importance of Motivational Beliefs

Another potential outcome of perceived positive social experiences, and also that of the enactment of psychological processes, is a perceived state of motivation (Franken, 2007). The study of motivation within the field of Educational Psychology is extensive, with different theoretical perspectives offered and research inquired undertaken. Maslow (1968, 1970) *humanistic theory of motivation*, for example, focuses on a person’s motive to achieve self-fulfillment of different needs (e.g., a need to self-fulfill and experience of love), whereas the *cognitive perspective of motivation* focuses on theoretical understanding of attribution (e.g., attribution of success that reflects one’s own personal ability) (Weiner, 1972, 1986), self-determination (Deci and Ryan, 1991, 2008), and value-expectancy beliefs (Eccles et al., 1983; Wigfield and Eccles, 2000). Researchers and educators have over the years proposed different inquiries for research development, focusing on antecedents and consequences of a state of motivation and, likewise, a state of demotivation. Research undertakings into the complex nature of motivation (Sansone and Harackiewicz, 2000; Pintrich and Schunk, 2002; Franken, 2007; Schunk et al., 2008) are effective and beneficial, providing theoretical insights and fruitful information for the purpose of practicality – for example, what is the most effective strategy to instill extrinsic motivation?

Our conceptualization of motivation, ξ_3_, as a distinct construct, depicts three comparable and interrelated constructs, interest in learning (X_1_), motivation toward learning (X_2_), and self-efficacy for learning (X_3_). The stipulation of X_1_, X_2_, and X_3_, in this case, enables us to provide a definition of motivation, which we consider it as being “a multifaceted and positive entity that encompasses one’s personal interest and a heightened state of self-belief to accomplish enriched learning experiences for different types of intrinsic reasons”. This consideration, for us conceptually, contends that the nature of motivation is intrinsic, wherein a person’s intent and purposive act is to learn and accomplish for intrinsic reasons, such as enjoyment, fulfillment of an inner desire to achieve mastery, and to improve personal self-belief of competence. From our proposition then, a person’s low level of interest and motivation, as well as his/her inefficacy for academic learning (i.e., a weakened state of self-efficacy) would undermine, weaken, and result in underperformance in a subject matter. Self-efficacy and high levels of interest and motivation, in contrast, would improve and/or facilitate a student’s learning experience.

There is a plethora of research that has yielded clear and consistent evidence, highlighting the potent role of motivation as a facilitator and predictor of different types of adaptive outcomes (Pintrich and Schunk, 2002; Franken, 2007; Schunk et al., 2008). What is innovative and relatively unique about our conceptualization, however, relates to the stipulation of contrasting predictive effects of both latent factor (i.e., ξ_3_ → O, where ξ_3_ = motivation, O = adaptive outcome, → = predictive effect) and measured indicators of motivation (e.g., X_1_ → O, where X_1_ = interest in learning, O = adaptive outcome, → = predictive effect) on different types of adaptive outcomes. For example, evidence of a positive effect of self-efficacy on achievement of an adaptive outcome would provide theoretical insights into the development of a teaching strategy and/or an educational program that could enhance and foster motivation, resulting improved academic performances and enriched learning experiences.

Our conceptualization also allows us to examine different types of antecedents that could account for the formulation of motivation, in general. What causes a student to feel motivated to engage in a particular course of action, academically and/or non-academically? In relation to self-efficacy (Bandura, 1997), for example, a number of correlational studies have attested to the effect of enactive learning experience such as successful accomplishments (Lent et al., 1991; Pajares et al., 2007; Liem et al., 2008; Usher and Pajares, 2008). In a similar vein, existing research has found numerous factors that could influence a person’s interest for learning and proactive engagement (Senko and Miles, 2008; Upadyaya et al., 2011; Walkington, 2013). We contend, as shown in Figure 1, that the two latent variables of perceived social experiences, ξ_1_, and psychological processes, ξ_2_, and their respective measured indicators could influence motivation and its respective measured indicators.

## Accomplishment of Adaptive Outcomes

Focusing on achieving and/or experiencing different types of adaptive outcomes in school contexts is more inclusive than the notion of “academic achievement.” Academic achievement, we contend, is relatively restricted, limiting to the seeking of understanding of a student’s academic learning and his/her accomplishment of learning outcomes. More recently, educators and researchers have advocated for the development of *holistic education* (Forbes, 2003; Hare, 2010), which focuses on the “totality” of a student’s schooling experiences – for example, cognition, social relationship, moral development, etc. Holistic education, in this sense, espouses the important viewpoint that educational successes do not simply mean and/or entail high academic grades, but rather encompass a myriad of school-based experiences (e.g., sound social development, and/or the fostering of emotional well-being) (Phan and Ngu, 2019a).

In terms of the present study, we conceptualize the concept of adaptive outcomes as a distinct latent factor, ξ_4_, which consists of three corresponding measured indicators: a student’s current best practice (i.e., what a student is capable of at present – for example: “I am able to understand and solve equations with one unknown, *x*, at present”) (X_1_) and optimal best practice (i.e., what a student perceives his/her maximum capability to be – for example: “I perceive that I am capable of solving equations with three unknowns, *x*, *y*, and *z*”) (X_2_) in a subject matter (Fraillon, 2004), as well as his/her perceived “academic liking experience” (X_3_) (Van Damme et al., 2002). This conceptualization of adaptive outcomes, we contend, is innovative as it does not place emphasis on actual test scores and/or academic grades, hence, helping to negate the academic pressure of a student having to perform well academically. Moreover, from our point of view, the inclusion of best practice is significant as it reflects recent development into the study of optimal best (Fraillon, 2004; Liem et al., 2012; Phan et al., 2019d; Phan and Ngu, 2021a). Optimal best is a positive concept, emphasizing a person’s state of flourishing or flourished experience in a subject matter. Indication of optimal best, aided by current best practice, entails the maximization in a person’s state of functioning (e.g., a person’s maximized state of cognitive functioning at the present time). On this basis, a person’s achievement of adaptive outcomes would showcase his/her positive academic liking experience (e.g., “I enjoy attending school”), as well as high levels of current and optimal best practice in a subject matter.

Aside from empirical evidence from existing research undertakings, we also use the *theoretical paradigm of philosophical psychology* (Thagard, 2014, 2018; Phan et al., 2020d) to assist us with our development of a conceptual framework for investigation. This theoretical paradigm reflects and entails the reliance and use of personal intuition, logical reasoning, philosophical understanding, and previous research development to conceptualize new ideas and viewpoints. For example, our recent research focus on the subject of *life and death education* (Chen, 2012, 2017; Huang, 2014) led to our use of philosophical reasoning and the proposed concept of “esoteric psychology” (Phan et al., 2020d, 2021c). In a similar manner, in tandem with extensive research studies reviewed so far, we use philosophical psychology as a basis to assist us with our conceptualization, which connotes the potency of three comparable direct effects on students’ adaptive outcomes, ξ_4_: perceived social experiences, ξ_1_, psychological processes of effective learning, ξ_2_, and motivational belief, ξ_3_. Philosophically, in this sense, a question that we could ask is whether there is credence to argue that ξ_1_, ξ_2_, and ξ_3_ would positively influence ξ_4_? At the same time, however, it is plausible for us to consider the direct predictive effects of the measured indicators of ξ_1_, ξ_2_, and ξ_3_ on ξ_4_ – for example: perception of the social milieu → adaptive outcomes versus personal resolve → adaptive outcomes. It is also likely that ξ_1_, ξ_2_, and ξ_3_ and their respective measured indicators could predict the three measured indicators of ξ_4_ – for example: perceived social experiences, ξ_1_, → current best practice versus perception of the social milieu → current best practice. Overall, our proposition for examination is insightful and could potentially provide valuable information for guidance, especially in terms of design and development of pedagogical practices, learning objectives, etc., that would enhance and improve different types of adaptive outcomes.

## Significance of the Present Study

Overall, then, the preceding sections have provided a rationalization of a conceptualization, which depicts a number of relationships for investigation. As shown in Figure 1 and Table 2, the significance of our research proposition lies in our attempt to test a structural model that places emphasis on two types of predictive relationship: predictive effects of latent factors and predictive effects of measured indicators. This consideration into two distinct “levels” or types of prediction is interesting, especially from a methodological point of view. Our conceptualization coincides with the nature of the paradigm of positive psychology (Seligman and Csíkszentmihályi, 2000; Seligman et al., 2009; Seligman, 2010), and reflects the positivity and proactivity of human agency – for example, academically, we postulate that positive psychological processes could predict and/or explain a student’s achievement and experience of optimal best in a subject matter. This focus into the proactivity and fostering of adaptive outcomes (e.g., a focus on optimal best) is different from the study of the negative or deficit nature of the schooling processes (e.g., a focus on school disengagement) (Liem et al., 2008; Tam, 2011; Henry et al., 2012), which would require remedy and the use of preventive measures to counter such discourse (e.g., how to prevent and negate negative schooling experiences). We contend that our proposed inquiry is significant as it highlights the potential interrelations between three distinct theoretical orientations (i.e., perceived social experiences versus psychological processes *versus* motivational beliefs), which then could account for a student’s adaptive outcomes.

**TABLE 2:** Latent theoretical concepts, definitions, and measured indicators.

**Latent theoretical concepts**	**Our definition**	**Measure indicators**
*Perceived social experiences*	A present perception (i.e., positive versus negative) of the myriad of different social experiences that a student may have at school or in university	• The Social Milieu
		• Relating to others
		• Pathways and means
*Psychological processes*	A student’s continuing frame of mind to focus on disposition toward strong resolute, structured thoughts and organization, and aspiration to strive for educational success	• Personal resolve
		• Effective functioning
		• Academic striving
*Motivation*	Motivation is multifaceted, global, and positive, encompassing personal interest and a heightened state of self-belief to accomplish enriched learning experiences for intrinsic reasons	• Interest in learning
		• Motivation toward learning
		• Self-efficacy
*Adaptive outcomes*	A person’s academic experience at school or in university (i.e., positive *versus* negative), as well as his indication of achievement of best practice in different subject matters	• Current best practice
		• Optimal best practice
		• Academic liking experience

## Materials and Methods

### Sample and Procedure

Seven hundred and thirty-nine undergraduate students (*N* = 442 women, 297 men) from three private universities in Taiwan participated in this study. The dataset, collected in 2019, forms part of our larger research project, involving secondary and university students from Australia, Malaysia, and Taiwan. The study reported in this manuscript was approved by the University of New England Research Ethics Committee. Because of the fact that the participants were all adults (i.e., over the age of 18), we decided to use a simpler approach for recruitment, which the university approved – that is, to verbally seek permission at the onset and anyone who did not want to take part to inform us. This method of verbally seeking participatory consent, used by us on previous research undertakings, is more convenient logistically.

Our sampling was convenient as it was logistically difficult to seek permission from students in other universities and colleges to take part in the present research study. Aside from this difficulty, limited resources also deterred us from attempting to expand on our data collection. The participants voluntarily took part in the study, knowing that there were no incentives. We administered the questionnaires in class, using the traditional paper-format. Overall, answering the Likert-scale measures took approximately 35–40 min to complete. The front page of the questionnaires contained demographic information – for example, which university a student attended, and what degree program he/she was studying. Overall, all the participants were full-time students and came from 40 different departments across the three universities.

The medium of formal instruction at school and in university in Taiwan is Chinese Mandarin. In 2016, we translated the questionnaires from English to Chinese Mandarin using a three-step methodological procedure: (i) Step 1: to translate the questionnaires from English to Chinese Mandarin (E_1_ → CM_1_), (ii) Step 2: to translate the translated questionnaires from Chinese Mandarin to English (CM_1_ → E_2_), and (iii) Step 3: compare the original English version of the questionnaires (i.e., E_1_) with the translated version (i.e., E_2_) (e.g., see Phan et al., 2019d for an in-depth description:). We have since then used the questionnaires with different cohorts, and results published (Phan et al., 2019c, d, 2020e) show sound psychometric properties (e.g., factorial structures).

### Instruments

Overall, from the preceding sections, there are 12 variables, which involved the use of 12 corresponding subscales, measured on a Likert-scale with five ratings – for example: 1 (Complete Not True), 2 (Not True), 3 (Neutral), 4 (True), and 5 (Completely True).

#### The Social Milieu

The Social Milieu Subscale (Phan and Ngu, 2016a; Phan et al., 2019c), consisting of five items – for example: “I find this university is very welcoming” and “This university is very accepting of people from other cultures.” Cronbach’s alpha value for the subscale upon examination was 0.77.

#### Relating to Others

We (e.g., Phan et al., 2019c) recently adapted the LOSO Questionnaire (Van Damme et al., 2002) and developed five items to measure and assess the concept of relating to others – for example: “I find it easy to relate to others (e.g., lecturers) at university” and “I find often it difficult to express my feelings to others (e.g., peers) at university.” Cronbach’s alpha value for the subscale upon examination was 0.74.

#### Pathways and Means

For consistency, awhile back, we developed five items to measure and assess the concept of pathways and means (Phan and Ngu, 2014). To date, we have not had an opportunity to use this subscale. The items included, for example: “I want to explore different options that are available to help me with my studies” and “I accept any help (e.g., utilization of resources) that is available to help with my studies.” Cronbach’s alpha value for the subscale upon examination was 0.76.

#### Personal Resolve

We adapted five items from recent research (Phan et al., 2018b, Phan et al., 2019d) to measure and assess the concept of personal resolve, which include, for example: “I will do whatever it takes to master my academic studies at university” and “I have a strong desire to succeed in my academic studies at university.” Cronbach’s alpha value for the subscale upon examination was 0.83.

#### Effective Functioning

We adapted five items from recent research (Phan et al., 2018; Phan et al., 2018b) to measure and assess the concept of effective functioning, which included, for example: “I have been told at university that I am quite efficient” and “I always keep to my routine when studying at university.” Cronbach’s alpha value for the subscale upon examination was 0.70.

#### Academic Striving

We developed five items to measure and assess the concept of academic striving, which included, for example: “I always strive to achieve good academic results at university” and “I see very little point in achieving high results at university.” Cronbach’s alpha value for the subscale upon examination was 0.77. Interestingly, with another cohort of university students in 2017, and recently published (Phan et al., 2020e), we achieved a reliability estimate of 0.78 for this subscale.

#### Interest in Learning Tasks

We adapted and used five items from the LOSO Questionnaire (Van Damme et al., 2002) to measure and assess the concept of interest in learning tasks. The items included, for example: “I enjoy learning the different subjects in this university” and “I believe many things we learn in university are not important.” Cronbach’s alpha value for the subscale upon examination was 0.81.

#### Motivation Toward Learning

We adapted and used five items from the LOSO Questionnaire (Van Damme et al., 2002) to measure and assess the concept of motivation toward academic learning. The items included, for example: “I can do much better for some academic subjects at university than I do now” and “I rarely do my best at university.” Cronbach’s alpha value for the subscale upon examination was 0.79.

#### Personal Belief of Efficacy for Learning

We adapted five items from the Motivated Strategies for Learning Questionnaire (Pintrich et al., 1991, 1993) to measure and assess the concept of personal belief of efficacy for academic learning. The items included, for example: “I believe I will receive excellent grades in classes at this university” and “I expect to do well academically in my classes for different subjects (e.g., Psychology).” Cronbach’s alpha value for the subscale upon examination was 0.82.

#### Current Best Practice

From theorization of best practice (e.g., Fraillon, 2004; Phan et al., 2018), we revised our original subscale, known as the Optimal Outcomes Questionnaire (Phan et al., 2015), which consisted of eight items, to a shorter version of five items – for example: “I am content with what I have accomplished so far at this university” and “I can achieve what is being asked of me at this university.” Cronbach’s alpha value for the subscale upon examination was 0.81. Previous research studies reported similar reliability estimates (e.g., Phan et al., 2018).

#### Optimal Best Practice

Similar to current best practice, we revised our original subscale (Phan et al., 2015) so that the new version has five items – for example: “I can achieve much more at university than I have indicated through my work so far” and “I want to learn and do more at university.” Cronbach’s alpha value for the subscale upon examination was 0.73.

#### Academic Liking Experience

We adapted the LOSO Questionnaire (Van Damme et al., 2002) and developed five items to measure and assess the concept of academic liking experience. The items included, for example: “I really like going to university” and “I would rather stay at home than to attend university” (Note: negative item). Cronbach’s alpha value for the subscale upon examination was 0.84.

## Data Analyses

**FIGURE 2:**
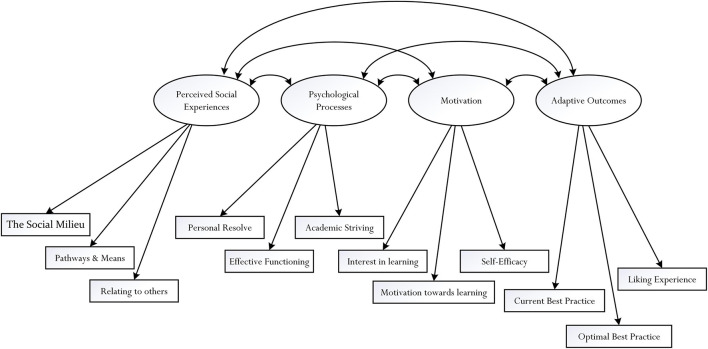
Factorial structure of perceived social experiences, psychological experiences, motivation, and adaptive outcomes. Each measured indicator (e.g., the Social Milieu) is made of a composite score of responses from the five items.

Our data analyses, overall, consisted of two main stages: (i) a four-factorial structure using confirmatory factor analysis (CFA) techniques (Bollen, 1989; Kline, 2011), and (ii) a complete structural model to validate our original *a priori* model (Figure 1), using structural equation modeling (SEM) techniques (Bollen, 1989; Kline, 2011). Overall, the participants responded to a suite of 60 Likert-scale items, which we then formed composite scores for the 12 measured indicators. In other words, each measured indicator (e.g., the Social Milieu measured indicator, the Relating to others measured indicator, the Pathways and Means measured indicator, etc.) is a composite score of five individual item responses. In sum, from this calculation, we have: 12 measured indicators × 4 latent factors (Note: each latent factor is defined by three measured indicators, and each measured indicator is made up of a composite score of five items – see Figure 2 for explanation.

### Confirmatory Factor Analysis

A four-factorial structure is interesting and may, in this case, provide evidence of factor loadings and the potential interrelations between the four latent factors. We used the statistical software package *MPlus 8.5* (Muthén and Muthén, 1998–2012) to assist with our analyses of a four-factor model. Likewise, SPSS 25 was used for descriptive statistics (e.g., data screening). Per guidance (Bollen, 1989; Kline, 2011), we performed an initial data screening test to ensure multivariate normality and the justification of using maximum likelihood (ML) estimates to test our hypothesized model [e.g., kurtosis values ranging from −0.06 (Academic Striving) to 1.02 (Optimal Best Practice), Std error = 0.18; skewness values ranging from −0.61 (Current Best Practice) to 0.06 (Academic Striving), Std error = 0.09]. From previous research undertakings (e.g., Green et al., 2012; Liem et al., 2012; Phan et al., 2019d), we considered the following indices to assist in the gauging of appropriate model fits: the *Comparative Fit Index* (CFI) (i.e., CFI value > 0.95), the *Tucker Lewis Index* (TLI) (i.e., TLI value > 0.95) the *Root Mean Square Error of Approximation* (RMSEA) (i.e., RMSEA value < 0.07), the *χ*^2^ test statistic, and an evaluation of parameter estimates were used in the present research to assess model fit (Liem and Martin, 2011).

Correlations between mean scale scores are shown in Table 3. Our CFA undertaking for the four-factor model showed an appropriate model fit, as indicated by the following goodness-of-fit index values: CFI = 0.94, TLI = 0.92, RMSEA = 0.08, *p* < 0.001 (Lo90 = 0.071, Hi90 = 0.090), and χ^2^/d*f* = 5.77. From Table 4, the factor loadings ranged from 0.52 to 0.77 for perceived social experiences, 0.70 to 0.75 for psychological processes, 0.63 to 0.70 for motivation, and 0.53 to 0.86 for adaptive outcomes, *p* < 0.001. associations also existed between the four latent factors: *r* = 0.95, *p* < 0.001 for psychological processes and perceived social experiences, *r* = 0.92, *p* < 0.001 for motivation and perceived social experiences, *r* = 0.89, *p* < 0.001 for motivation and psychological processes, *r* = 0.77, *p* < 0.001 for adaptive outcomes and perceived social experiences, *r* = 0.82, *p* < 0.001 for adaptive outcomes and psychological processes, and *r* = 0.77, *p* < 0.001 for adaptive outcomes and motivation.

**TABLE 3:** Correlations between means scale scores.

	**Mean (SD)**			**Milieu**		**Relate**		**Path**		**Resolve**		**Effect**		**Striving**		**Interest**		**Moti**		**Efficacy**		**Current**		**Optimal**		**Like**

	**Total**	**Women**	**Men**																							
*1*	3.69 (0.70)	3.77 (0.66)	3.58 (0.74)	1.00																						
*2*	3.72 (0.72)	3.81 (0.67)	3.60 (0.77)	0.41	**	1.00																				
*3*	3.86 (0.58)	3.89 (0.53)	3.81 (0.63)	0.39	**	0.46	**	1.00																		
*4*	4.04 (0.64)	4.06 (0.62)	4.00 (0.67)	0.30	**	0.35	**	0.57	**	1.00																
*5*	3.64 (0.66)	3.69 (0.63)	3.56 (0.70)	0.35	**	0.42	**	0.61	**	0.45	**	1.00														
*6*	3.75 (0.69)	3.79 (0.65)	3.68 (0.75)	0.31	**	0.45	**	0.52	**	0.57	**	0.53	**	1.00												
*7*	3.48 (0.71)	3.55 (0.68)	3.38 (0.73)	0.34	**	0.37	**	0.48	**	0.42	**	0.39	**	0.42	**	1.00										
*8*	3.43 (0.71)	3.56 (0.65)	3.23 (0.74)	0.28	**	0.30	**	0.40	**	0.39	**	0.42	**	0.49	**	0.50	**	1.00								
*9*	3.76 (0.74)	3.85 (0.71)	3.62 (0.75)	0.42	**	0.51	**	0.48	**	0.42	**	0.43	**	0.52	**	0.45	**	0.40	**	1.00						
*10*	3.98 (0.75)	4.08 (0.70)	3.84 (0.81)	0.16	**	0.28	**	0.34	**	0.46	**	0.31	**	0.36	**	0.27	**	0.28	**	0.29	**	1.00				
*11*	3.80 (0.67)	3.87 (0.63)	3.69 (0.71)	0.33	**	0.43	**	0.50	**	0.57	**	0.43	**	0.49	**	0.43	**	0.41	**	0.45	**	0.47	**	1.00		
*12*	3.49 (0.61)	3.58 (0.58)	3.36 (0.63)	0.33	**	0.43	**	0.48	**	0.50	**	0.45	**	0.46	**	0.42	**	0.41	**	0.48	**	0.37	**	0.70	**	1.00

**Correlation is significant at the 0.01 level (2-tailed).

*(1) Milieu, Social Milieu; (2) Relate, relating to others; (3) Path, pathways and means; (4) Resolve, personal resolve; (5) Effect, effective functioning; (6) Striving, academic striving; (7) Interest, interest in learning; (8) Moti, motivation toward learning; (9) Efficacy, personal self-efficacy; (10) Current, current best practice; (11) Optimal, optimal best practice; (12) Like, academic liking experience.*

**TABLE 4:** Factor loadings for a four-factor model.

	**Perceived Social Experiences**	**Psychological Processes**	**Motivation**	**Adaptive Outcomes**
*Social Milieu*	0.52	–	–	–
*Relating to others*	0.63	–	–	–
*Pathways and means*	0.77	–	–	–
*Personal resolve*	–	0.72	–	–
*Effective functioning*	–	0.70	–	–
*Academic striving*	–	0.75	–	–
*Interest in learning*	–	–	0.67	–
*Motivation toward learning*	–	–	0.63	–
*Self-efficacy*	–	–	0.70	–
*Current best practice*	–	–	–	0.53
*Optimal best practice*	–	–	–	0.86
*Academic liking experience*	–	–	–	0.81

*Factor loadings are statistically significant at *p* < 0.001.*

### Structural Equation Modeling

The results established from the factorial structure analysis substantiated our SEM undertakings, which consisted of a baseline model. Overall, from the hypothesized *a priori* model shown in Figure 1, there are four latent factors (i.e., perceived social experiences, psychological processes, motivation, and adaptive outcomes) and 12 measured indicators. The base-line model, denoted as Model M_0_, consisted of six structural paths for statistical testing: the structural path from perceived social experiences to psychological processes, the structural path from psychological processes to motivation, the structural path from perceived social experiences to motivation, the structural path from motivation to adaptive outcomes, the structural path from psychological processes to adaptive outcomes, and the structural path from perceived social experiences to adaptive outcomes. The results of this base-line model, using covariance matrices as correlation matrix analysis is known to entail potential problems (e.g., producing incorrect goodness-of-fit index values (Byrne, 1998; Jöreskog and Sörbom, 2001) are relatively modest in terms of fit – for example: χ^2^/d*f* = 5.77, CFI = 0.94, TLI = 0.92, RMSEA = 0.080. From a researcher’s point of view, existing theorizations and research development (e.g., Schumacker and Lomax, 2004; Kline, 2011; Byrne, 2012) would recommend the finalization and discussion of the results of Model M_0_. Having said this, we are interested to test different iterations of Model M_0_, via modification fit indices.

We are interested, in this case, to explore the relationships between both latent factors and measured indicators. Statistical software packages such as *MPlus 8.5* (Muthén and Muthén, 1998–2012) provide modification fit indices to assist a researcher in his/her quest to achieve an optimal fit for *an a priori* model. Having said this, researchers have cautioned the use of modification fit indices, contending that respecification of an *a priori* model depends on rationale and/or strong theoretical and/or empirical grounding (Byrne, 1998; Kline, 2011). Our testing and comparison of different *a priori* and *a posteriori* models is interesting, as it enables us to establish theoretical understanding into L_1_ (e.g., perceived social experiences → psychological processes) and L_2_ (e.g., relating to others → psychological processes) relationships. Table 5 shows the testing of seven *a posteriori* models. Goodness-of-fit index values are also provided to help finalize and determine the optimal fit model for discussion.

**TABLE 5:** Summary of fit-index values for models tested.

**Model**	**Description of model**	**χ_2_**	**d*f***	**Δχ_2_**		**CFI**	**TLI**	**RMSEA**		
									**L090**	**Hi90**
								
M_0_	Baseline model with the following paths: Perceived social experiences → Psychological processes Psychological processes → Motivation Perceived social experiences → Motivation Motivation → Adaptive outcomes Psychological processes → Adaptive outcomes Perceived social experiences → Adaptive outcomes	277.08	48	–		0.94	0.92	0.080	0.071	0.090
M_1_	Model M_0_ with the inclusion of: Pathways and means → Effective functioning	236.04	47	Δ_(*M*0–*M*1)_ = 41.04	***	0.95	0.93	0.074	0.065	0.083
M_2_	Model M_1_ with the inclusion of: Relating to others → Self-efficacy	202.68	46	Δ_(*M*1–*M*2)_ = 33.36	***	0.96	0.94	0.068	0.058	0.078
M_3_	Model M_2_ with the inclusion of: Personal resolve → Adaptive outcomes	177.94	45	Δ_(*M*2–*M*3)_ = 24.74	***	0.96	0.95	0.063	0.054	0.073
M_4_	Model M_3_ with the inclusion of: Pathways and means → Personal resolve	145.73	44	Δ_(*M*3–*M*4)_ = 32.21	***	0.97	0.96	0.056	0.046	0.066
M_5_	Model M_4_ with the inclusion of: Personal resolve → Current best practice	121.39	43	Δ_(*M*4–*M*5)_ = 24.34	***	0.98	0.97	0.050	0.039	0.060
M_6_	Model M_5_ with the inclusion of: Self-efficacy → Academic liking experience	109.42	42	Δ_(*M*5–*M*6)_ = 11.97	***	0.98	0.97	0.047	0.036	0.057
M_7_	Model M_6_ with the inclusion of: Academic striving → Motivation toward learning	105.07	41	Δ_(*M*6–*M*7)_ = 4.35	*	0.98	0.97	0.046	0.035	0.057

*p < 0.05, ***p < 0.001.

The respecification of a base-line model and, subsequently, a comparison of two competing models (e.g., Model M_0_ versus Model M_1_) require the use of the Δχ^2^ test, as well as an inspection of the goodness-of-fit index values. The *principle of parsimony*, in this case, advocates for the acceptance of a less restricted model. Overall, as shown in Table 5, the results (e.g., the use of the Δχ^2^ test as well as various goodness-of-fit index values) indicate a progression in improvement of model fit from Model M_0_ to Model M_7_. From the modification fit index values, we respecified Model M_0_ to include seven additional structural paths, which we freed one at a time:

(i) Model M_1_: the inclusion of the path from the measured indicator, pathway and means, to the measured indicator, effective functioning. This structural path (β = 0.33, *p* < 0.001) places emphasis on the provision of opportunities, pathways, and means, which would encourage and/or compel a student to be more structured in his thinking, organization, and/or planning – for example, an educator informing to the class that there are limited resources available, requiring students to be more effective. This example is logical and suggests that, despite opportunities, pathways, and means, circumstances and personal situations made instill understanding of effective functioning.
(ii) Model M_2_: the inclusion of the path from the measured indicator, relating to others, to the measured indicator, self-efficacy. This structural path (β = 0.20, *p* < 0.001) substantiates Bandura (1997) social cognitive theory, placing emphasis on the use of vicarious information to instill a heightened state of self-efficacy. There is evidence, in this analysis, to support and affirm the importance of socially derived information (e.g., proactive peer social relationship in class) for the purpose of self-efficacy (Usher and Pajares, 2006; Phan, 2012).
(iii) Model M_3_: the inclusion of the path from the measured indicator, personal resolve, to the latent factor, adaptive outcomes. This structural path (β = 0.28, *p* < 0.001) is interesting, highlighting the L_2_ → L_1_ relationship. Moreover, evidence of this structural path supports recent development into the predictive and explanatory of personal resolve (e.g., Phan et al., 2018; Phan et al., 2020e), highlighting the importance of a person’s state of unwavered concentration and decisiveness. A high level of personal resolve, in this analysis, may mobilize persistence and compel a student to expend more effort, resulting in accomplishment of different types of adaptive outcomes.
(iv) Model M_4_: the inclusion of the path from the measured indicator, pathways and means, to the measured indicator, personal resolve. This structural path (β = 0.25, *p* < 0.001) is significant, highlighting the significance of pathways and means – in this analysis, similar to that of effective functioning, the provision of opportunities, pathways, and means is beneficial, enabling a student to develop skills and experiences to be decisive in her academic studies. For example, opportunities to engage in complex learning tasks, and/or the use of encouraging and persuasive feedbacks may instill personal resolute to succeed.
(v) Model M_5_: the inclusion of the path from the measured indicator, personal resolve, to the measured indicator, current best practice. This structural path (β = 0.22, *p* < 0.001), importantly, supports previous evidence that affirms the predictive nature of personal resolve (e.g., Phan et al., 2018; Phan et al., 2020e). Interestingly, however, the direct effect of personal resolve is insightful for the purpose of theoretical contribution to the nature of current best practice. For example, a state of resolute and decisiveness may play a poignant role in helping a student to make more accurate judgments and assessments of his/her existing capability (e.g., “This is what I am capable of at present”).
(vi) Model M_6_: the inclusion of the path from the measured indicator, self-efficacy, to the measured indicator, academic liking experience. This structural path (β = 0.12, *p* < 0.01) support existing research development, which affirms the potent effect of personal self-efficacy for academic learning (e.g., Pajares and Miller, 1994; Bandura, 1997; Liem et al., 2008; Martin et al., 2010). A heightened state of self-efficacy, for example, is likely to mobilize various cognitive and non-cognitive processes (e.g., persistence in the face of difficulty), which would result in learning and other school-based accomplishments (e.g., academic buoyancy) (Martin et al., 2010).
(vii) Model M_7_: the inclusion of the path from the measured indicator, academic striving, to the measured indicator, motivation toward learning. This structural path (β = 0.11, *p* < 0.01) is significant, supporting recent research development into the predictive nature of the concept of academic striving (Phan et al., 2020e). Academic striving, based on the paradigm of positive psychology (Csíkszentmihályi, 1990; Seligman, 1999; Seligman and Csíkszentmihályi, 2000), is conceptualized as a positive and proactive concept (e.g., seeking to strive for a future endeavor in Astronomy is a positive feat), which would act to instill confidence and motivation. Indicating an inner desire to strive for educational success, for example, is likely to result in a state of motivation (e.g., “I am motivated to work hard in order to fulfill my goal”).

**FIGURE 3:**
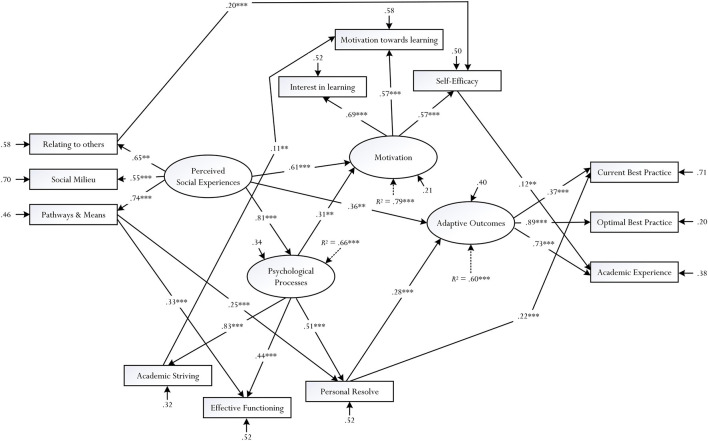
Final ***solution of model*** M_7_. ^∗^***p*** < 0.05, ^∗∗^***p*** < 0.01, ^∗∗∗^***p*** < 0.001. Non-statistically significant paths have been omitted for clarity.

As shown in Table 5, the Δχ^2^ tests showed an improvement in model fit, progressively, from Model M_0_ to Model M_7_. Aside from the Δχ^2^ tests, an inspection of the goodness-of-fit index values also supported the testing of different iterations of Model M_0_, and the acceptance of a less restrictive model. Overall, from analyses of different *a posteriori* models, we accept the results of Model M_7_ for discussion (e.g., χ^2^/d*f* = 2.56, CFI = 0.98, TLI = 0.97, RMSEA = 0.046). The solution of Model M_7_ is depicted in Figure 3 and, indeed, shows some interesting evidence in terms of the associations between the four latent factors: (i) that perceived social experiences positively influenced psychological processes (β = 0.81, *p* < 0.001), motivation (β = 0.61, *p* < 0.001), and adaptive outcomes (β = 0.36, *p* < 0.01), (ii) the positive effect of psychological processes on motivation (β = 0.31, *p* < 0.01), and (iii) that both psychological processes and motivation did not influence adaptive outcomes. Furthermore, of the four latent factors, evidence established indicated the potentiality for psychological processes to as a central mediator between perceived social experiences and motivation: the total effect from perceived social experiences on motivation is 0.87, *p* < 0.001 in which the decomposition is follows: direct effect of perceived social experiences on motivation is 0.61, *p* < 0.001, whereas the direct effect, mediated via psychological processes is 0.26, *p* < 0.01.

## Discussion of Results

A focus on effective learning and, more importantly, the proactivity of human agency within the context of academic learning is a positive endeavor for accomplishment. How do we promote mastery and/or exceptional academic performance in educational contexts? Is there an overarching system that could explain and/or account for students’ effective learning and/or enriched schooling experiences? Numerous conceptualizations and research undertakings have provided comprehensive theoretical overviews, detailing comparative insights into the intricacy of the teaching and learning processes. One interesting line of inquiry, spanning the course of more than two decades relates to the positive psychology of a person, organization, and community – a state of flourishing (Keyes and Haidt, 2003; Seligman, 2010; Huppert and So, 2013), for example, is positive and reflects the enactment of the psychological processes of motivation and proactivity of a person, which may situate within his/her sociocultural system of change.

Our scholarly research interest has led to the development of a conceptual framework, which we explored in the present quantitative study. One pervasive line of inquiry that we considered, in this case, is whether and/or to what extent contrasting explanatory and predictive effects of both latent factor and measured indicator levels could positively influence different types of adaptive outcomes. In summary, evidence that we have obtained from SEM analyses is insightful, making theoretical, empirical, and methodological contributions to the study of optimal learning and schooling experiences. Figure 3, interestingly, illuminates a number of statistically significant pathways, or trajectories, which account for improvement of a student’s academic learning experience. Such pathways, we contend, are informative in helping educators to design and develop different types of educational and non-educational programs for implementation.

### Theoretical Contributions of the Present Study

An inspection of Figure 3 indicates a number of pathways, or trajectories, that are statistically significant, accounting *R*^2^ = 0.60 for the concept of adaptive outcomes, which is made up of a student’s current best practice, optimal best practice, and academic experience. An important question that we often ask, as educators, is how we would encourage and foster students’ educational experiences in school or university contexts. One possible approach to this is to consider pathways, opportunities, and/or means that could assist to improve, strengthen, and/or facilitate different students’ learning and non-learning experiences. Interestingly, as summarized below, there are a number of statistically significant pathways:

(1) *Predictive effects at the latent factor level:*
  The effect of perceived social experiences on psychological processes.
  The effect of perceived social experiences on adaptive outcomes.
  The effect of perceived social experiences on motivation.
  The effect of psychological processes on motivation.
(2) *Predictive effects at the measured indicator level:*
  The effect of relating to others on self-efficacy.
  The effect of pathways and means on effective functioning.
  The effect of pathways and means on personal resolve.
  The effect of academic striving on motivation toward learning.
  The effect of personal resolve on current best practice.
  The effect of personal resolve on adaptive outcomes^∗^.
  The effect of self-efficacy on academic striving.

^∗^Outcome is a latent concept – that is, a measured indicator → latent concept, where → = predictive effect.

From the above, there are five notable findings that are of significance: (i) the role of perceived social experiences as an important antecedent of psychological processes, motivation, and adaptive outcomes, (ii) the positive effect of psychological processes on motivation, (iii) the central role of psychological processes as a predictor as well as a mediator (Baron and Kenny, 1986; Trafimow, 2015), (iv) explanatory accounts and predictive effects of both latent factors (e.g., perceived social experiences → psychological processes) and measured indicators (e.g., relating to others → self-efficacy) level, and (v) empirical validation of a four-factor structure, depicting factor loadings (e.g., psychological processes consisted of personal resolve, effective functioning, and academic striving) and interrelations between the four latent factors.

What we can conclude from the established findings, as detailed in the preceding sections? There are three overarching aspects for consideration, which may advance our theoretical understanding into the positivity and proactivity of human agency. One achievement of human agency in educational contexts, in this case, relates to a student’s autonomy and motivation to achieve optimal learning experiences (Phan et al., 2019b). The conclusion derived from SEM analyses contends the following:

(i) *The importance of perceived social experiences*: The positive effect of perceived social experiences (e.g., perception that there are pathways and means for growth) coincides with existing research development (Flook et al., 2005; Mansouri and Kamp, 2007; Roorda et al., 2011) and, in this case, depicts the importance of the social contexts at hand (Vygotsky, 1978; Bronfenbrenner, 1989; Lave and Wenger, 1991). For example, in a recent study, Eraslan-Capan (2016) reported that a student’s perceived social connectedness (e.g., how one understands and views his/her closeness with others) positively influenced his/her state of flourishing. Interestingly, too, Flook et al. (2005) study found that negative social experiences (e.g., a student’s perceived lack of peer acceptance) actually weakened both academic performance and academic self-concept. Our findings, in tandem with existing evidence, indicate that perceived social experiences, situated within different sociocultural contexts, play a notable role (e.g., helping a child to socially adjust) (Roorda et al., 2011).
(ii) *Psychological sub-processes of learning*: The positive effect of psychological processes on motivation supports our acknowledgment of the importance of positive psychology (Seligman and Csíkszentmihályi, 2000; Seligman et al., 2009; Seligman, 2010), as a distinct paradigm, in the teaching and learning processes. Interestingly, however, the explanatory account of psychological processes (e.g., psychological processes → motivation) reflects our proposition of its underlying nature – namely that it is consisted of three sub-processes, which have previously been examined: academic striving, effective functioning, and personal resolve. What can we draw from this finding? That in terms of accounting for motivation and/or an improvement in schooling experiences, we could consider the instilment and/or enactment of academic striving, effective functioning, and/or personal resolve. For example, evidence of the predictive role of personal resolve (e.g., personal resolve → current best practice) is consistent with existing research inquiries (e.g., Phan et al., 2018b; Phan and Ngu, 2021a), which show a positive effect of this psychological concept on different types of adaptive outcomes (e.g., task-specific self-efficacy belief for academic learning). The positive effect of academic striving on motivation toward learning, similar to previous research (Phan et al., 2020e; Phan and Ngu, 2021a), is significant, highlighting the positive nature of this psychological concept. Personal striving to accomplish a specific goal, regardless of whether one is able or not, in this sense, may act as a source of motivation, directing and motivating a person to actively engage in the learning process.
(iii) *Motivation*: Motivation did not statistically influence adaptive outcomes; rather, and interestingly, self-efficacy positively influenced academic liking experience and, likewise, personal resolve influenced adaptive outcomes. This evidence (i.e., self-efficacy → academic experience) supports and coincides with existing research studies (Pajares and Miller, 1994; Liem et al., 2008; Martin et al., 2010; Phan et al., 2018b), which emphasize the explanatory power of self-efficacy for academic learning (Bandura, 1977, 1997). There are two plausible reasons, however, as to why we did not find an association between the latent motivation factor and the latent adaptive outcomes factor. Firstly, there is a potential constructive “misalignment” between the two latent factors, commonly denoted as “motivation ≠ adaptive outcomes.” Past research studies, interestingly, have alluded to a theoretical tenet known as the specificity and contextualization of motivation (Pajares, 1996b; Bandura, 1997; Phan et al., 2019d). Specificity and contextualization, in this case, emphasize the notion of microanalytical assessment and, more importantly, a close correspondence between psychological and/or achievement-related variables under examination. Secondly, our SEM analyses were somewhat “exploratory,” which could in effect resulted in the testing of other iterations – for example, the removal of the direct path from the latent perceived social experiences factor to the latent adaptive outcomes factor (i.e., fixing this path to 0) could have resulted in a statistically significant path from the latent motivation factor to the latent adaptive outcomes factor.

### Practical Implications for Consideration

The present study, aside from empirical and theoretical contributions, has also provided enriching insights for the purpose of daily relevance and practical application. This acknowledgment recognizes the importance of the nexus between research and quality teaching and learning experiences. Our focus of inquiry, differing from a deficit approach (e.g., a focus on school failure or task disengagement) (Liem et al., 2008; Henry et al., 2012), is significant for its emphasis on the proactivity and positivity of human behavior. From the perspective of schooling, in general, it is a valuable feat to focus on the promotion and encouragement of enriched student experiences, academically and/or non-academically – for example, a secondary school student may attend and enjoy school for various reasons (e.g., to partake in extracurricular activities), or a university student who enjoys her learning and seeks to engage in mastery. There are a number of educational implications that are of value for us to consider. For the purpose of conciseness, we have included Table 6, which surmises our construction of different propositions and recommendations for consideration.

**TABLE 6:** A summary of educational considerations.

**Recommendations for consideration**	**Purpose**

**Perceived social experiences**	
• Focus on providing in-class opportunities for different types of growth (e.g., problem solving) – for example: exposing students to different challenging learning tasks and/or activities, which would encourage social interactions and, hence, discussions, debates, and collaborative learning	To encourage social interactions and, hence, relatedness, friendship, academic assistance, where appropriate
• Encourage and foster a positive institutional social climate for different types of growth – for example: encourage students to take part in different types of mentoring programs (e.g., the “Buddy Program” where a postgraduate student may mentor a group of 4th-year undergraduate students)	To encourage leadership and, more importance, social relatedness between students for different reasons – for example, social support, emotional security, and academic scaffold To foster a positive and proactive social milieu for enriched learning experiences

**Psychological processes**	

• Expose students to different metacognitive strategies (e.g., the use of reflective journals to record daily learning experiences)	To teach and prepare students to be more organized in their planning, organization, and reflection of learning, which emphasizes the importance of effective functioning
• Provide critical, but encouraging and positive feedbacks (e.g., “You are not doing this correctly, Chou; You need to be more purposive here…. yes, excellent!”)	To teach and prepare student to be more decisive, determined, and unwavered in their decision making, which reflects the importance of personal resolve
• To purposively fail students and, hence, from this, to have students experience failures in their academic learning experiences	To teach and encourage students to “bounce back” from their failures. This experience, from our point of view, reflects the importance of personal resolve
• Encourage students to consider and/or to record a 5-year future time plan – for example, what would they realistically like to accomplish in 5 years’ time?	To encourage students to have aspirations for fulfillment, regardless of whether these are achievable or not. This point emphasizes the importance of academic striving for success

**Motivation**	

• Provide encouraging feedback (e.g., “This is great, Monica! See if you can continue on with……”) in a timely manner	To instill confidence and, more importantly, personal self-efficacy for learning
• Situate and design subject contents to emphasize more on mastery rather than performance-based learning experiences, which would encourage competition	To encourage and instill intrinsic motivation for learning
• Situate and design subject contents to reflect authenticity with life-related relevance, if possible	To encourage and instill interest for learning at university

An analysis of the propositions and recommendations shown in Table 6 indicates one commonly theme – namely, to consider the use of effective pedagogies, programs, institutional policies, etc., that could promote the development of psychosocial (e.g., positive social relationship at school) and/or psychological experiences (e.g., a heightened state of personal resolve), which in turn would improve engagement of different types of adaptive outcomes. Moreover, Table 6 is significant for its depiction of practical emphasis, which may differ in nature and varieties – for example, the use of verbal discourse (e.g., encouraging feedback) as opposed to exposing students to different types of metacognitive strategies. Aside from this description, what else can we consider for practicality? Recently, for example, our research inquiries have delved into the topical theme of *life and death education* (Chen, 2009, 2012; Huang, 2014; Phan et al., 2020c) from the perspective of Taiwanese Education. One aspect of life and death education, reflecting both quality teaching and research development, is the incorporation of Buddhist teaching (Yeshe and Rinpoche, 1976; Master Sheng, 2010; Thanissaro, 2015), which may encompass Buddhist mindfulness and meditation practice (e.g., “walking meditation”). The underlying premise of Buddhist teaching, in accordance with the study of life and death education, is related to the notion of “spiritual cultivation” (Phan et al., 2020c, 2021c). Buddhist meditation, in this sense, may instill and cultivate a “purified mindset,” directing and assisting a person to feel more enlightened and spiritual. An interesting question then is whether and/or to what extent a spiritual mindset, via means of engagement in Buddhist meditation, say, could promote and/or predict a person’s psychological processes (e.g., a person’s heightened state of personal resolve).

### Methodological Consideration and Future Directions

The present study has provided some important methodological insights into the measurement and assessment of psychosocial and psychological concepts. One interesting aspect of our undertaking involved the use of Likert-scale measures, situated within a non-experimental context. Non-experimental data, especially cross-sectional are extremely restricted, limiting a researcher from making casual inference and/or personal inference (Rogosa, 1979; MacCallum and Austin, 2000). By all account, the use of self-reporting is inadequate in terms of providing robust and stringent information, which could capture and/or illustrate the underlying nature and intricacy of internal psychological processes. Self-reporting is descriptive and, in this case, provides evidence of perception, judgment, feeling, and belief, all of which may indicate inaccuracy. Pajares and his colleagues researching the topic of self-efficacy for academic learning in the 1990s alluded to an interesting phenomenon, which they termed as “miscalibration” (e.g., Pajares and Miller, 1994; Pajares and Kranzler, 1995; Pajares, 1996a). Miscalibration, in this case, considers two contrasting phenomena: a state of overconfidence *versus* a state of underconfidence. In the context of the present study then, it is plausible to contend that a university student may miscalibrate and report, for example, a high level of personal resolve or effective functioning. In a similar vein, a secondary school student may have some form of grievance toward his teacher, resulting in a biased report that indicates very little, if any, academic and/or social support at school (i.e., a biased indication of the indicator of pathways, means, and opportunities). On this basis, we argue for the use of alternative methodological designs, which could address the aforementioned shortcomings.

An interesting observation that we note, which has potential methodological relevance relates to *in situ* observations may complement the use of “meditative reflection” and subsequent self-reporting of personal feelings and experiences (Phan et al., 2021c). Meditative reflection and self-reporting, as a whole, is internal and arises from within a student (i.e., *subjective* in nature), whereas a teacher’s *in situ* observation of the student is external (i.e., *objective* in nature). This consideration of using multiple strategies of data collection, we contend, is more effective as this could provide a balanced, well-rounded viewpoint and/or testament (i.e., subjective *versus* objective) of a person’s behavior, psychological thoughts, emotions, experiences, etc. As such, contrasting methodological strategies may add credence to assist in the validation and elucidation of a process, relationship, etc.

We posit that the hypothesized relationships depicted in Figure 1 and/or the obtained solution shown in Figure 2 are somewhat complex and as such, as we previously mentioned, using cross-sectional and non-experimental data is inadequate. We encourage the use of longitudinal data as these could offer a more robust methodological approach in validating and/or substantiating long-term flow and personal growth. As an example, which may support our rationale, the underlying nature of academic striving (e.g., Phan and Ngu, 2020a, 2021a; Phan et al., 2020e) is complex and would, in this instance, require longitudinal data for accurate assessment and theoretical understanding. When a person aspires and strives to achieve an optimal state of functioning (Fraillon, 2004; Liem et al., 2012; Phan et al., 2016c), he/she would require time, effort, the availability of resources, etc. As such, in terms of appropriateness, we advocate for the use of multiple time points in data collection as this longitudinal consideration would offer a logical and more accurate capture of a student’s personal resolve, striving, etc., – for example, a student may indicate to us the following (T_1_): “I want to strive and achieve Honors in Psychology by the time I graduate” (Date of indication, for example: 13th May, 2020). The question then is whether this mentioning of aspiration and personal striving would eventuate in 2 years’ time, when the student would have completed her academic studies (T_2_).

Finally, one of our reviewers provided some interesting methodological insights, which we deem may play a critical role in future research development. The study of perceptions (e.g., a perception of positive social experience *versus* negative social experience), especially when it comes to diversity and sampling requires formal recognition and acknowledgment of potential selection bias and limitations. For example, as the reviewer highlights, our participants came from different universities and departments and consequently, from this variation and/or diversity, their individual differences were not well controlled. Thus, from this observation of “dissimilar” backgrounds, we urge readers to read and interpret our results with caution. In a similar vein, of course, our own observation recognizes that the sample was relatively modest in size, limiting us from undertaking additional and/or different types of statistical analysis – for example, a statistical undertaking, which involves a *factorial variance/invariance* analysis, identifying potential equivalency (Byrne, 1998) between different groupings (e.g., the extent to which the final solution depicted in Figure 3 is invariant across both men and women). A larger sample size, situated within hierarchical clusters or structures (e.g., universities × faculties × schools × departments), likewise, may provide opportunities for multilevel modeling (Little, 2000).

## Conclusion

The present study tested a conceptual model, which considered three distinct but interrelated theoretical orientations within a system of change: psychosocial influences, positive motivational beliefs, and psychological processes. Established evidence via means of correlational analysis is of significance, detailing specific pathways that could account for an improvement in a person’s adaptive outcomes. The statistical techniques of SEM, interestingly, offer logical grounding for the analysis and study of associations between both latent (e.g., the positive effect of psychological processes on motivation) and measured indicator (e.g., the positive effect of self-efficacy on academic experience) levels. Aside from empirical, theoretical, and methodological contributions, our research undertaking also provided sound, meaningful insights for educational purposes.

## Data Availability Statement

The original contributions presented in the study are included in the article/supplementary material, further inquiries can be directed to the corresponding author.

## Ethics Statement

The studies involving human participants were reviewed and approved by the University of New England Research Ethics Committee. Written informed consent for participation was not required for this study in accordance with the national legislation and the institutional requirements.

## Author Contributions

HP and BN contributed equally to the conceptualization, articulation, and writing of the manuscript. Both authors contributed to the article and approved the submitted version.

## Conflict of Interest

The authors declare that the research was conducted in the absence of any commercial or financial relationships that could be construed as a potential conflict of interest.

## Publisher’s Note

All claims expressed in this article are solely those of the authors and do not necessarily represent those of their affiliated organizations, or those of the publisher, the editors and the reviewers. Any product that may be evaluated in this article, or claim that may be made by its manufacturer, is not guaranteed or endorsed by the publisher.
